# How Demanding Is Volunteer Work at a Crisis Line? An Assessment of Work- and Organization-Related Demands and the Relation With Distress and Intention to Leave

**DOI:** 10.3389/fpubh.2021.699116

**Published:** 2021-07-15

**Authors:** Renate C. W. J. Willems, Constance H. C. Drossaert, Harald S. Miedema, Ernst T. Bohlmeijer

**Affiliations:** ^1^Research Center Innovations in Care, Rotterdam University of Applied Science, Rotterdam, Netherlands; ^2^Department of Psychology, Health and Technology, University of Twente, Enschede, Netherlands

**Keywords:** crisis line volunteers, stressors, demands, intention to leave, distress, mental wellbeing

## Abstract

**Background:** Crisis line services, run by volunteers, offer a listening ear 24/7 to people who cannot or do not want to use professional help. Although previous studies have identified various potential stressors crisis line volunteers face, as yet a comprehensive assessment is lacking with regards to the frequency and perceived stressfulness of work- and organization-related demands, and their relationship with distress and a volunteer's intention to leave.

**Objective:** To identify the frequency and impact of particular stressful situations (demands). In addition, to examine the extent to which these demands are associated with volunteers' demographics, distress and intention to leave the crisis line service.

**Method:** In a cross-sectional study among 543 volunteers of a Dutch crisis line service the participants filled out a questionnaire about their experience of a large number of work- and organization-related demands and their perceived stressfulness. To calculate the impact of demands, the occurrence and stressfulness were multiplied. In addition, work-related distress, intention to leave the crisis line service as well as a number of demographics and work-related characteristics were assessed.

**Results:** Work-related demands with the highest impact on volunteers were calls from people with psychiatric problems and suicidal intentions. “Having no time for a break” was the organization-related demand with the highest impact on volunteers. Eighteen percentage of the volunteers scored moderate or high on distress and 4% had the intention to leave the crisis line service within 1 year. Most work- and organization-related demands were positively associated with volunteers' distress and intention to leave the organization. Being older, being male and spending more hours per week volunteering were significantly, positively correlated with work-related demands. The total explained variance for distress was 16% and for intention to leave 13%.

**Conclusion:** Although most crisis line volunteers experienced low impact from work- and organization-related demands, these demands were significantly related to experienced distress and the intention to leave crisis line service. For volunteers with moderate to high distress it can be useful to implement interventions aimed at increasing personal resources to help them deal with the challenges of the work at the crisis line.

## Introduction

“*The telephone rings. A man says he's injected a very high dose of insulin and he's going to die soon. But he doesn't want to be alone in the last moments of his life, so he asks the crisis line volunteer if she wants to be with him in the last moments of his life. The volunteer and the caller talk about the life and approaching death of the man.”*

“*After this impressive conversation, the phone rings again. The caller is hard to understand, he seems to have been drinking. After asking a few times what the caller says, the caller starts to curse. He screams that there are a bunch of idiots on the phone, which are of no use to him at all. Angry, he ends the call.”*

“*A chat comes in: ‘I'm so worried about my mom. She has Alzheimer's and she lives alone. I don't think it's safe and I want her to go to a nursing home. The doctor doesn't think this is necessary, and the waiting lists are very long. I can't handle the care anymore; nobody wants to listen to me.' The volunteer listens and asks questions. During the conversation the caller calms down and thinks for herself who she can approach for practical support.”*

“*A new chat is coming in. Someone, calling himself John, says he's having a hard time. Six months ago, he was fired at work and his partner decided to leave him. The volunteer recognizes the story, this chatter chats several times a week, each time with the same story”*

*(Cases described by crisis line volunteers during an interview)*.

Crisis line services, an important addition to existing formal care, provide free, emotional support 24/7 by telephone, chat, or email (1). This form of low-threshold support gives callers^1^ quick anonymous and confidential access to emotional support, sometimes until professional care is available (2, 3). Crisis line services are mostly operated by volunteers, who are recruited and trained to provide a non-judgmental, active listening service, which invites callers to reflect on their suffering and emotional distress in order to understand how they can cope with their problems (1). Crisis line services are effective in preventing suicidality (4, 5) and reducing caller distress (5, 6). Although volunteers at the crisis line service are well-trained, volunteering can be demanding. Several studies have shown that the demands of the work at the crisis line may impact volunteers' mental wellbeing (7, 8) and their intention to leave the crisis line organization (9). However, research on the demands faced by volunteers working at a crisis line service is scarce. A few qualitative studies revealed a number of demands related to the work itself (characteristics of callers or the topics) and a number of demands related to the organization of the work.

The literature highlights that crisis line volunteers most commonly face three work-related demands that are potential challenges to volunteering. Firstly, the topics of the calls, such as suicidality or abuse experiences (5, 10). A qualitative study revealed that these complex topics may generate feelings of powerlessness, sadness, and shock among crisis line volunteers (Willems et al., submitted). Topics that are not directly related to suicidality and violence, such as loneliness, relationship problems, and boredom (11, 12), may also be experienced as stressful by the volunteers. In many cases volunteers perceive such conversations to be trivial and that their time may be better used on “real” problems (11). Secondly, the inappropriate behavior of certain callers. This includes callers who try to get sexual satisfaction during the conversation (13, 14), callers who discriminate or scold and are insulting toward the volunteer, or callers who try to get personal information from the volunteer (15). Thirdly, the so-called “frequent callers”: callers who call the crisis line several times a day, often with the same story (16–18). Frequent callers are not time-wasters, but they do keep the telephone line busy. Pirkis et al. (18) showed in a literature review that only 3% of the callers are frequent callers, but they are responsible for 60% of incoming calls. Balancing between the great diversity of different conversation topics and dealing with difficult behavior of clients requires great mental flexibility from the volunteer, which can influence their motivation to continue doing this work, or can lead to distress or leaving the job (7, 8).

Organizational-related demands have to do with the way the work is organized, and may also be perceived as a challenge by the volunteers. For example, most crisis line services use the philosophy of non-intervention, which means that volunteers only offer a listening ear, not a therapeutic intervention (9). The philosophy of non-intervention may give volunteers the feeling that they are not allowed to offer any concrete solution to the caller, while they do want to take action (9). Another organization-related challenge is the policy of anonymity, which means that callers can anonymously tell their problems to volunteers. As a result, volunteers will never know whether their conversation has been effective, or what influence this conversation had on the caller. This can create a feeling of insecurity among the volunteers (15, 19, 20). Inadequate support and backup (e.g., through supervision, training) and the length of shifts are further examples of organization-related demands (9, 20–22).

Studies on work- and organizational-demands among crisis line volunteers have often been conducted with small samples, or they are qualitative in design. Moreover, most studies have focused on a single or a few work- and organization-related demands, rather than studying them in a more comprehensive way. Therefore, it remains unclear to what extent these demands occur and are perceived as stressful. Such insight is important, because previous studies have shown that crisis line volunteers can suffer from high work-related distress, and that traumatic experiences can be a reason for quitting their volunteer work (9, 20, 23).

It is important to study the factors associated with the occurrence and perceived stressfulness of the various demands, in order to better understand why some crisis line volunteers experience more stress at work than others. Previous research has provided some evidence for a relationship between demographic and work-related factors (such as years of experience and former education) and mental wellbeing of crisis line volunteers. For example, younger age (21, 23, 24), less experience at the crisis line (21, 23), and being female (23) have shown to be positively correlated with stress. However, research on the issue is scarce, and there is a lack of knowledge about the relationship between demographic and work-related variables on the one hand and distress, and the impact of particular demands on the other hand.

The aim of this study is to identify the occurrence and stressfulness of a wide range of work- and organization-related demands among crisis line volunteers, and to determine to what extent there is a relationship with distress of the volunteers and their intention to leave working at the crisis line service. The questions that will be answered are: (a) What is the perceived frequency and stressfulness of various work- and organizational demands that crisis line volunteers experience? (b) Which demands are most strongly associated with volunteers' distress and intention to leave? (c) To what extent are the impact of demands, volunteers' distress and intention to leave associated with volunteers' demographics (gender, age, education) and work-related variables (years of experience, number of hours a week)?

## Materials and Methods

### Design

The present study used a cross-sectional design with an online survey among crisis line volunteers. The study was approved by the ethical committee of the Faculty of Behavioral and Management studies (BMS) of University of Twente (approval number: 190943).

### Participants and Procedure

The crisis line volunteers were recruited from the “Listening Line,” a Dutch crisis line service, run by 1,400 volunteers who are trained to be non-judgmental, empathetic, respectful and caring. The “Listening Line” applies the principle of non-intervention, this means that no therapeutic intervention is applied, volunteers are only lending an ear (1, 25).

All crisis line volunteers of “the Listening Line” (*n* = 1,405) received a link to the questionnaire by email from their management. The respondents were given an explanation of the survey and an online informed consent form. After the respondents had given their consent, they could continue to complete the anonymous questionnaire. After 2 and 4 weeks a reminder was sent by email. The questionnaire was completed by 543 volunteers (response rate 39%).

### Measures

#### Personal Background Variables

Demographic characteristics included age and gender. Regarding their work at the crisis line, we asked participants if they had a professional training in health care (for example social work, nursing, or psychology), the number of years of experience at the crisis line, the number of hours per week at the crisis line, and from which location they mostly conducted their volunteer work (from the crisis line service office, or from home). For an overview of the wording of all questions and response options, see Table 1.

**Table 1:** Demographics and work-related information by gender.

		**Total (*****N*** **=** **543)**		**Male (*****N*** **=** **155)**		**Female (*****N*** **=** **387)**	
		**Freq**.	**Percent**	**Freq**.	**Percent**	**Freq**.	**Percent**
Age	18–29	10	1.8	3	1.9	7	1.8
	30–49	38	7.0	7	4.5	31	8.0
	50–64	200	36.8	46	29.7	154	39.8
	>65	294	54.1	99	63.9	195	50.4
Professional training in healthcare	Yes	196	36.1	42	27.1	154	39.8
	No	347	63.9	114	73.5	233	60.2
Experience at the crisis line	<1 year	105	19.3	37	23.9	68	17.6
	1–3 years	193	35.5	57	36.8	136	35.1
	3–6 years	89	16.4	24	15.5	65	16.8
	6–10 years	58	10.7	16	10.3	42	10.9
	>10 years	98	18.0	22	14.2	76	19.6
Hours per week	<4 h per week	97	17.9	22	14.2	75	19.4
	4–6 h per week	408	75.1	119	76.8	289	74.7
	6–8 h per week	31	5.7	14	9.0	17	4.4
	8–10 h per week	4	0.7	0	0.0	4	1.0
	>10 h per week	3	0.6	1	0.6	2	0.5
Location of work	Always on location	133	24.5	53	34.2	80	20.7
	Usually on location, occasionally at home	91	16.8	30	19.4	61	15.8
	Sometimes on location, sometimes at home	55	10.1	17	11.0	38	9.8
	Usually at home, occasionally on location	126	23.2	28	18.1	98	25.3
	Always at home	138	25.4	28	18.1	110	28.4

#### Work-Related and Organization-Related Demands

##### Specific Demands

A self-developed questionnaire was used to measure demands that are specific for crisis line work. The items were based upon results from a literature review (8) and a qualitative study among volunteers of “the Listening Line” (Willems et al., submitted). The questionnaire consisted of two parts: work-related demands (16 items) and organization-related demands (9 items). Each item describes a potentially distressing situation that a crisis line volunteer may encounter (see Tables 2, 3 for an overview of items). For 20 (of the 25) demands two questions were asked: the first question related to the occurrence of the situation [“How often does this situation occur?” with answering options ranging from “never” (0) to “very often” (4)] and the second question related to the degree of stress that this situation causes [“How stressful is this situation for you?” answering options ranging from “not at all stressful” (1) to “very stressful” (5)]. For the remaining five demands the frequency question could be answered with no (1) and yes (2) and the degree of stressfulness was measured on a 5-point scale as described above. The *impact* of each demand was calculated by multiplying the frequency of occurrence with the degree of stress produced by the demand. This calculation was only applied to the 20 questions that could be answered with the five-point scale. For the other five questions the degree of stress was considered as the impact.

**Table 2:** Summary of descriptive statistics work-related demands: frequency, degree of stress, and impact (product frequency and degree of stress)^*^ and Spearman's rho correlations with Intention to Leave (ITL) and Distress (*N* = 543).

	**Occurrence**				**Stressfulness**	**Impact**	**Correlation with:**	
**Possible range**	**1/2**	**3**	**4/5**	**1–5**	**1–5**	**1–25**	**ITL**	**Distress**
	**Never/Sometimes**	**Regularly**	**Often/Very often**	**Mean (SD)**	**Mean (SD)**	**Mean (SD)**	**r_**s**_**.	**r_**s**_**
	**%**	**%**	**%**					
Client has psychiatric problems; is confused, agitated, or gloomy	14	45	41	3.4 (0.9)	1.9 (0.8)	6.4 (3.8)	0.19^**^	0.22^**^
Client is suicidal	82	15	3	2.2 (0.6)	2.8 (1.1)	6.2 (2.9)	0.12^**^	0.20^**^
Client manipulates, scolds, discriminates, shocks, judges, or seeks quarrel	86	13	1	2.1 (0.5)	2.5 (1.1)	5.3 (2.8)	0.14^**^	0.22^**^
Client is talking so much that volunteer can't intervene; speech waterfall	35	50	15	2.8 (0.8)	1.8 (0.9)	5.3 (3.5)	0.14^**^	0.21^**^
Client complains and whines	36	46	18	2.8 (0.8)	1.8 (0.9)	5.2 (3.4)	0.13^**^	0.26^**^
Client puts the problem with the volunteer, adopts a passive attitude, and assumes the victim role	51	40	10	2.6 (0.8)	1.9 (0.9)	5.0 (3.3)	0.16^**^	0.18^**^
Client doesn't listen, thinks in extremes	56	3	10	2.5 (0.7)	1.9 (0.9)	4.9 (3.2)	0.11^**^	0.27^**^
Client has sexual intentions with the conversation	79	18	2	2.2 (0.6)	1.9 (1.1)	4.4 (3.1)	0.11^**^	0.15^**^
Client tells story in which children or animals are victims	92	7	1	1.8 (0.6)	2.3 (1.2)	4.3 (2.6)	*ns^*a*^*	0.23^**^
Client calls several times a day with the same story	41	45	14	2.8 (0.8)	1.5 (0.8)	4.2 (2.8)	0.16^**^	0.21^**^
Client has a life-threatening or serious physical illness	77	20	3	2.2 (0.6)	1.8 (0.9)	4.1 (2.3)	*ns*	*ns*
Client is under the influence of alcohol or drugs and cannot communicate properly	84	15	2	2.1 (0.5)	1.7 (0.9)	3.6 (2.3)	*ns*	0.21^**^
Client is busy with other things during conversation	89	10	1	2.0 (0.6)	1.8 (0.9)	3.6 (2.6)	*ns*	0.26^**^
Client tells a bizarre story that's probably not true	71	26	3	2.3 (0.6)	1.5 (0.7)	3.4 (1.9)	0.13^**^	0.14^**^
Client presents physical complaints, while in fact there are psychological problems	61	33	6	2.4 (0.7)	1.3 (0.6)	3.2 (1.9)	0.11^**^	0.14^**^
Client says he intends to mistreat someone (human or animal)	99	1	0	1.3 (0.5)	2.2 (1.4)	3.0 (2.3)	*ns*	0.16^**^
**Scale score “Work-related demands” (α** **=** **0.88)**						**4.4 (1.6)**	0.24^**^	0.33^**^

* *Items are ordered by impact. highest impact at the top;*

** *Correlation is significant at the 0.01 level (2-tailed);*

a *ns is not significant. The test in bold shows the correlation with ITL and distress of the total scale work-related demands*.

**Table 3:** Summary of descriptive statistics organization-related demands: frequency, degree of stress, and impact (product frequency and degree of stress)^*^ and Spearman's rho correlations with Intention to Leave (ITL) and Distress (*N* = 543).

	**Occurrence**				**Stressfulness**	**Impact**	**Correlation with:**	
**Possible range**	**1/2**	**3/4**	**4**		**1–5**	**1–25**	**ITL**	**Distress**
	**Never/ sometimes**	**Regularly**	**Often/ very often**					
	***%***	***%***	***%***	**Mean (SD)**	**Mean (SD)**	**Mean (SD)**	**r_**s**_**.	**r_**s**_**
Volunteer hardly have time for a break	50	24	26	2.6 (1.3)	1.3 (1.0)	5.2 (4.4)	*ns*^a^	0.20^**^
There is little contact with other volunteers/employees because they work from home	54	20	26	2.6 (1.3)	1.3 (0.6)	3.5 (2.9)	0.22^**^	0.18^**^
Organization does not listen carefully to wishes or needs of volunteer/employee	92	5	3	1.5 (0.8)	1.5 (0.9)	2.5 (3.1)	0.11^**^	0.18^**^
The support teams not accessible, although there is a need for it	100	0	0	1.1 (0.4)	1.3 (0.8)	1.5 (1.5)	*ns*	*ns*
	**No**	**Yes**			**Degree of stress**		**ITL**	**Distress**
	**%**	**%**			**1–5 Mean**		**Corr**.^**b**^	**Corr**.^**b**^
The organization applies the philosophy of non-intervention	–				2.0 (1.1)	–	ns	0.20^**^
Volunteer must work night shifts	26	74			2.0 (1.2)	–	0.20^**^	0.15^**^
The shifts are too long	75	25			1.5 (0.8)	–	0.18^**^	0.18^**^
The client is anonymous, therefore the volunteer does not know what effect the conversation has had	–				1.5 (0.8)	–	*ns*	0.19^**^
The location of the telephone helpline is not optimal	88	12			1.3 (0.7)	–	*ns*	0.16^**^

* *Items are ordered by impact, highest impact at the top;*

** *Correlation is significant at the 0.01 level (2-tailed);*

a *ns is not significant;*

b *Correlation between degree of stress and ITL/Distress*.

An exploratory factor analysis was carried out regarding the work-related demands. It appeared that all items loaded on a single factor. A Cronbach's α coefficient of 0.88 was obtained for the current sample, indicating an excellent internal consistency. Since the organization-related demands showed very low or double factor loads, no further factor analyses were conducted regarding organization-related demands. Due to the low α (0.53) the organization-related demands could not be combined into one scale.

### Outcome Variables

#### Intention to Leave

Intention to leave (ITL) was measured with a single-item question: “How likely is it that you will leave the crisis line service the coming year?” and could be scored on a five-point Likert scale ranging from “very unlikely” (1) to “very likely” (5).

#### Distress

Distress was measured with a subscale of the well-validated Four-Dimensional Symptom Questionnaire (4DSQ) (26). Distress is operationalized as reactions to stress, such as worry, irritability, tension, listlessness, poor concentration, sleeping problems and demoralization (27). This subscale contains 16 items that can be scored on a five-point Likert scale, ranging from 1 (never) to 5 (always). A Cronbach's α coefficient of 0.90 was obtained for the current sample, indicating excellent internal consistency. The occurrence of distress was determined by reducing the five answer categories of the Likert scale to three answer categories (never = 0, sometimes = 1, regularly or more often = 2), and subsequently summing the items to a total score, ranging from 0 to 32. Based upon these scores, participants were categorized into low (0–10), moderately increased (11–20), or strongly increased distress (21–32), as outlined in the 4DSQ manual (27).

### Analysis

Analyses were performed in Statistical Package of the Social Sciences (SPSS), version 26.0. Background variables and work-related variables were described using descriptive analysis. In order to determine the presence of demands and the degree of stress they cause, descriptive statistics were used. To identify the association between the impact of the demands and demographics on “intention to leave” and “distress” Spearman's correlation coefficients were computed, because of non-normality of intention to leave and distress. Hierarchical stepwise multiple linear regression analyses were conducted to examine the combined influence of demographics, work- and organization-related demands on distress and intention to leave.

## Results

### Sample and Descriptives

The majority of the participants were female and older than 50 years of age (see Table 1). Most of them had no professional training in health care, worked for 4–6 h a week at the crisis line services and had 1–3 years of experience in working as a crisis line volunteer. In terms of age and gender, the sample corresponded to the total population (1).

A total of 474 respondents (82%) scored low, 79 (15%) scored moderate, and 17 (3%) scored high on distress. Of all respondents, 81% indicated that it is very unlikely or unlikely that they will leave the crisis line service within a year, 16% indicated they “might leave” the crisis line service within a year, and 4% indicated they “were likely” to leave the crisis line service within a year.

### Impact of Work-Related and Organization-Related Demands and Their Relation to Intention to Leave and Distress

Table 2 shows the results on frequency and perceived stressfulness of the work-related demands. The most frequent work-related demands were conversations with clients who were confused, agitated or gloomy due to psychiatric problems and with clients who were whining and complaining. The most distressing work-related demands were clients with suicidal intentions or clients who are confused, agitated or gloomy due to psychiatric problems. When looking at the impact (combining frequency and perceived stressfulness), we see that clients with psychiatric problems and clients who were suicidal scored highest. The combined scale of work-related demands was significantly associated with intention to leave the crisis line service (*r*_*S*_= 0.24). The correlations between the impact of the separate work-related demands and intention to leave were generally weak, ranging from insignificant to *r*_*S*_= 0.19 (Table 2). The item “client has psychiatric problems, is confused, agitated, or gloomy” had the strongest correlation with intention to leave (*r*_*S*_ = 0.19). The combined scale of work-related demands was moderately associated with volunteers' distress (*r*_*S*_= 0.33). The correlations between the impact of separate work-related demands and distress are slightly higher than those of intention to leave, ranging from insignificant to *r*_*S*_= 0.27 (Table 2). The item “client is not listening and thinks in extremes” had the highest correlation with distress (*r*_*S*_= 0.27), followed by “client is complaining and whining” (*r*_*S*_= 0.26), or “client is doing other things during the conversation” (*r*_*S*_ = 26).

Table 3 shows the frequency, perceived stressfulness, and impact of organization-related demands. The most frequently occurring organization-related demands were: night shifts, hardly having time for a break and duration of shifts, with, respectively, 74, 50, and 25% of the volunteers saying that these situations were often occurring. The stressors that caused the highest level of stress were the philosophy of non-intervention and having to do night shifts. The stressor with the highest impact was not having time for a break. The correlations of the items of the organization-related demands with intention to leave and distress, are also shown in Table 3. Four out of nine demands were significantly associated with the intention to leave, with “little contact with co-workers, due to working from home” having the highest correlation (*r*_*S*_ = 0.22), followed by “having to do night shifts” (*r*_*S*_ = 0.20). Eight items were positively correlated with distress, with “The organization applies the philosophy of non-intervention” and “hardly having time for a break” having the highest correlations (*r*_*S*_ = 0.20).

### Relation Between Demographics and Work-Related Variables and the Impact of Demands

Older volunteers and volunteers who worked more hours per week reported significantly less impact of work-related demands (Table 4). Other demographic and work-related variables showed no significant relation with the perceived impact of work-related demands.

**Table 4:** Correlations between variables and means, and standard deviations of the scale variables.

		**Intention to leave**	**Distress**	**Work-related demands**
	**Possible range**	**1–5**	**0–32**	**0–20**
	**Mean (SD)**	**4.1 (0.9)**	**6.3 (5.5)**	**4.5 (1.6)**
1	Age	ns^d^	−0.14^*^	−0.21^*^
2	Gender^a^	ns	ns	0.12^*^
3	Professional training in health^b^	ns	ns	ns
4	Years of experience at the crisis line	ns	ns	ns
5	Hours per week at the crisis line	−0.20^*^	ns	−0.13^*^
6	Location of work^c^	ns	ns	ns

* *Correlation is significant at the 0.01 level (2-tailed)*.

a *1 = male, 2 = female*.

b *1 = yes, 2 = no*.

c *1 = always on location, 5 = always at home*.

d *ns is not significant*.

Volunteers with more years of experience reported significantly more impact from not having time for a break (*r*_s_ = 0.16), and significantly less impact from having to do night shifts (*r*_s_ = −0.22). Older volunteers experienced significantly less impact from working long shifts (*r*_s_ = −0.17) and night shifts (*r*_s_ = −0.19). Volunteers who worked more hours per week experienced significantly less impact from working long shifts (*r*_s_ = −0.13). Female volunteers experienced significantly more impact from working night shifts (*r*_s_ = −0.22). Other demographic and work-related demands showed no significant relation with the perceived impact of organization-related demands.

### Demographics and Demands as Determinants for Volunteers' Distress and Intention to Leave?

A summary of the multiple regression analysis of the determinants of distress is shown in Table 5. Demographics and work-related variables explained 4% of the variance of distress. Age, professional training in health, and years of experience in crisis line services were significantly associated with volunteers' distress. When the scale with work-related demands was added, the total amount of explained variance increased to 13%. By adding the organization-related demands, the total explained variance increased significantly to 16%. In particular the items: “There is little contact with other volunteers/employees because they work from home” and “The caller/chatter is anonymous, therefore the volunteer does not know what effect the conversation has had” added significantly to the explanation of volunteers' distress.

**Table 5:** Summary of multiple regression analysis of determinants of distress (*N* = 543).

**Model**	**Predictor**	**B**	**SE B**	**β**	
1^a^	Age	−0.08	0.02	−0.15^***^	*R^2^* = 0.04, *F*_(6, 536)_ = 4.08^**^
	Professional training in health	1.01	0.49	0.09^*^	
	Years of experience at the CLS	0.41	0.18	0.10^*^	
2^b^	Age	−0.04	0.02	−0.09^*^	*R^2^* = 0.13, *F*_(7, 5.356)_ = 11.30^***^
	Professional training in health	1.01	0.47	0.09^*^	
	Work-related demands	0.99	0.14	0.30^***^	
3^c^	Professional training in health	1.09	0.47	0.10^*^	*R^2^* = 0.16, *F*_(16, 526)_ = 6.44^***^
	Work-related demands	0.59	0.18	0.18^***^	
	. little contact with other volunteers/employees	0.19	0.08	0.10^*^	
	… lack of insight into effectiveness because of anonymity	0.69	0.33	0.10^*^	

* *p < 0.001,*

** *p < 0.01*,

*** *p < 0.05*.

a *Predictors: Age, Gender, Professional training in health, Years of experience at the CLS, Hours per week working at the CLS, Location of work*.

b *Predictors: Age, Gender, Professional training in health, Years of experience at the CLS, Hours per week working at the CLS, Location of work, Work-related demands*.

c *Predictors: Age, Gender, Professional training in health, Years of experience at the CLS, Hours per week working at the CLS, Location of work, Work-related demands, Organization does not listen carefully to wishes or needs of volunteer/employee, There is little contact with other volunteers because they work from home, The support team is not accessible, although there is a need for it, Volunteer hardly has time for a break, The organization applies the philosophy of non-intervention, The identity caller/chatter is anonymous, therefore the volunteer does not know what effect the conversation has had, The shifts are too long, Volunteer/employee must work night shifts, The location of the telephone helpline is not optimal*.

Table 6 shows a summary of the multiple regression analysis of the determinants of intention to leave the crisis line service. Demographics only explained 4% of the variance. Hours per week working at the crisis line service was the only significant characteristic that explained 4% of the variance of intention to leave. When the scale work-related demands was added, the total amount of explained variance increased to 8%. When organization-related demands were added, the total explained variance increased significantly to 13%. In particular the items: “There is little contact with other volunteers/employees because they work from home” and the item “The identity of the caller/chatter is anonymous, therefore the volunteer does not know what effect the conversation has had” added significantly to the explanation of volunteers' intention to leave.

**Table 6:** Summary of multiple regression analysis of determinants of intention to leave (*N* = 543).

**Model**	**Predictor**	**B**	**SE B**	**β**	
1^a^	Hours per week at the crisis line	−0.31	0.07	−0.2^*^	*R*^2^ = 0.04, *F*_(5, 537)_ = 4.67^*^
2^b^	Hours per week at the crisis line	−0.28	0.07	−0.19^*^	*R*^2^ = 0.08, *F*_(6, 536)_ = 7.37^*^
	Work-related demands	0.29	0.07	0.19^*^	
3^c^	Hours per week at the crisis line	−0.31	0.07	−0.2^*^	*R*^2^ = 0.13, *F*_(15, 527)_ = 5.18^*^
	…little contact with other volunteers	0.13	0.06	0.09^***^	
	… lack of insight into effectiveness because of anonymity	0.12	0.05	0.11^***^	
	…must working night shifts	0.1	0.03	0.14^**^	

* *p < 0.001*,

** *p < 0.01*,

*** *p < 0.05*.

a *Predictors: Age, Professional training in health, Years of experience at the CLS, Hours per week working at the CLS, Location of work*.

b *Predictors: Age, Gender, Professional training in health, Years of experience at the CLS, Hours per week working at the CLS, Location of work, Work-related demands*.

c *Predictors: Age, Gender, Professional training in health, Years of experience at the CLS, Hours per week working at the CLS, Location of work, Work-related demands, Organization does not listen carefully to wishes or needs of volunteer/employee, There is little contact with other volunteers because they work from home, The support team is not accessible, although there is a need for it, Volunteer has hardly time for a break, The organization applies the philosophy of non-intervention, The identity caller/chatter is anonymous, therefore the volunteer does not know what effect the conversation has had, The shifts are too long, Volunteer/employee must work night shifts, The location of the telephone helpline is not optimal*.

## Discussion

### Main Results

This is the first study to examine comprehensively which specific demands volunteers of a crisis line service are confronted with, how often these demands occur, how stressful they are and what impact they have on the volunteers' degree of distress and their intention to leave the crisis line service. In general, it can be concluded that some work-related demands are experienced as highly stressful but do not occur often (including callers who are suicidal and callers who tell stories in which children or animals are victims). Other demands are experienced as less stressful, but are quite common (including clients with psychiatric problems and frequent callers). The work-related demands with the highest impact (combined frequency and stressfulness) are callers with psychiatric problems, followed by callers who are suicidal. Yet, the demands that were most strongly associated to volunteer's distress were callers who do not listen and think in extremes, callers who complain and whine, and callers who are busy doing other things during the conversation.

Of all included stressors, callers with psychiatric problems scored highest on perceived impact, mostly because this stressor occurs often: 86% of the volunteers indicated being regularly or often called by people with psychiatric problems. In recent years, the Listening Line has detected an increase in conversations with psychiatric patients who, due to cutbacks in psychiatric care, received no (or less) treatment from their professional therapist (28). In 2018, one-third of conversations at the Dutch Listening Line were conducted with (former) clients from the mental health care sector (29). Other studies, conducted in Ireland and Iran, indicate that ~25% of callers have psychiatric problems (30, 31), which is less than callers at the Dutch Listening Line. In our previously mentioned qualitative study (Willems et al., submitted) some volunteers reported a sense of powerlessness with callers with psychiatric problems, because they are not able to alleviate their problems. However, other volunteers indicated that offering a listening ear to callers with psychiatric problems, made their work experience more meaningful resulting in feelings of gratitude and satisfaction. We recommend preparing new volunteers at crisis lines for the potentially high number of callers with psychiatric problems and to pay attention to the feelings of powerlessness that may arise as a result.

In the Netherlands, there is a separate crisis line for callers with suicidal ideations: 113-suicide prevention (32). As a result, callers with suicidal ideations are not common: in our study 82% of the volunteers indicate that they rarely or never have contact with people with suicidal ideations. However, when encountered, calls discussing suicidal ideations are (very) stressful. Targeted training and guidance in dealing with callers with suicidal ideations is necessary to maintain the wellbeing of volunteers. Future research could focus on the experienced stress among volunteers who work at a telephone helpline aimed at callers with suicidal ideations. This could provide input for training in dealing with this target group.

“Frequent callers” were less common than could be expected based on previous research. In our study only 14% indicated that they often or very often were confronted with frequent callers. Previous studies have found that frequent callers represent about 3% of callers, but 60% of calls (18, 33). Although most volunteers do not experience these frequent callers as stressful, they can cause some frustration and irritation (34, 35). Attention during supervision for dealing with frequent callers can help to reduce frustration and irritation.

As organization related demand, the lack of opportunities to have contact with other volunteers was mentioned quite often (46%). Although this demand was not perceived as very stressful, it was (of all organization-related demands) the most strongly associated with intention to leave. Lack of contact with co-volunteers is caused by the fact that many volunteers (49%) are working from home most of the time. As a result, volunteers may experience a lack of support or social contact. Yet, it should be noted that for some volunteers the connection with other volunteer workers is the main motivation to do the volunteer work (21, 36, 37). Lack of contact with co-volunteers also leads to fewer opportunities to talk about experienced stress and deal with difficult situations (19). It can even lead to a feeling of isolation among crisis line volunteers (20). Several studies conducted during the Covid-19 pandemic have shown that working from home and the lack of contact with colleagues can lead to distress (38, 39). Even though this is not entirely comparable to the sample of this study (volunteers can choose to work from home or from the office), it is indicative for the value of contact with co-volunteers. It is important that the organization provides opportunity for working at a common office where volunteers can meet. Also peer support activities should be encouraged to allow volunteers to share their work experiences (40).

Although the correlations between work-related demands and distress were generally low, some work-related stressors were more strongly associated with volunteers' distress: “callers who do not listen and think in extremes” (*r* = 0.27), “callers who are complaining and whining” (*r* = 0.26), and “callers who are busy doing other things during the conversation” (*r* = 0.26). It is striking that these situations showed the highest correlations with distress, because as far as we know, they were not previously described in the literature. A possible explanation for the relatively high impact of these stressors may be found in the motivation of volunteers to do this work. Volunteers working at a telephone helpline often have a strong motivation to make a difference by offering meaningful support (21, 36, 37, 41). Conversations with callers who are not open for support may be considered less meaningful and therefore less rewarding for crisis line volunteers. The issue of callers who are not open for support can be addressed in supervision meetings. Volunteers could be trained to help these callers to formulate their needs, or to accept that for some callers, complaining and whining can also be a relief. In further research into the contribution of stressors to reduction of well-being in crisis line volunteers, these demands should be included.

Only 18% of the respondents scored moderate or high on distress. This is lower than in other studies, where about 30% of crisis line volunteers scored moderate to high on general distress (22, 23). The low score on distress in all studies at crisis line volunteers can possibly be explained by the fact that respondents are volunteers, who have the possibility to stop volunteering at the crisis line when they experience high distress. Because former volunteers are not included in this study, we cannot make any statements about the level of distress among them when leaving this volunteer work. Further research on volunteers who have stopped volunteering will provide important additional information on the impact of working at the crisis line service on volunteers' mental wellbeing. Those who continue to work at the crisis-line, and are thus in our sample, may be the people with high personal resources, such as self-compassionate thinking and behavior that may prevent them from getting distressed. Another explanation for the relatively low score on distress in this study could be the available resources at the Dutch Listening Line, including good training, guidance, and supervision. At the Dutch Listening Line, potential volunteers receive extensive training, aimed at practicing difficult conversations. In addition, in the first months that they work as a volunteer, they receive guidance from a mentor, which is an experienced crisis line volunteer. Experienced volunteers also receive supervision, in which experiences with other volunteers can be exchanged (29). Information about the positive influence of resources can help to explain why these volunteers score low on distress so that other organizations can learn from this. Further research could also focus on this type of resources.

The total of explained variance for distress was 16%. Only 4% was explained by professional training in health and being older, which has also been demonstrated earlier (23, 24). In this study, no association was found between gender and the degree of distress. Another study showed that women experienced a higher degree of distress than men (23). Other causes of distress could come from personal events outside the crisis line service, or low perception of organizational and personal resources. This is beyond the scope of this study.

The low total of explained variance of intention to leave (13%) may reflect the fact that volunteers could have many other reasons than negative experiences to leave the crisis line service, such as moving, changing jobs, training, or babysitting grandchildren. However, these variables were not included in this study. To understand why the explained variance by work-related stressors on intention to leave is low, further (qualitative) research into the reasons why people have the intention to stop volunteering is required.

### Strengths and Limitations

This is the first study that provides insight into the occurrence and perceived stressfulness of a wide range of demands and their relationship with distress and intention to leave. The sample size of this study was much larger than other studies and was representative for the population in terms of age and gender. The design of our questionnaire (work-related demands) was based on a systematic review (8) and a qualitative study (Willems et al., submitted) and showed good internal consistency. The factor analysis showed that all items of the work-related demands loaded on one single factor and the instrument scored excellently on reliability. Therefore, the questionnaire can be used in further research into work-related demands among crisis line volunteers.

This study also has some important limitations: First, the study design is cross-sectional, which makes it impossible to establish a causal relationship between demands and intention to leave or distress. The second limitation is that the questionnaire has only been completed by respondents who are still volunteering at the crisis line service. It is possible that people who have experienced a lot of distress through volunteering at the crisis line service, already have stopped this voluntary work and are therefore not in the sample.

### Conclusion

Our study shows that many volunteers at a crisis line service experienced some distress, but one in seven reported moderate to high distress. Callers who are difficult to reach and who are not open to support are most strongly associated with volunteers' distress. Having little time for a break and adopting the philosophy of non-intervention are the organization-related demands most strongly associated with distress. Having little contact with fellow volunteers is most strongly correlated with intention to leave. Since volunteers are essential for the continuity of the crisis line services, it is of great importance that the organization management pays attention to these demands through training and supervision, in order to support distressed volunteers in adopting effective coping skills. In addition, it is important that the organization prepares potential volunteers for the topics they will face; and regularly organizes peer support activities aimed at connecting volunteers and share their work-experiences. Further research into the role of organization-related and personal resources can help explain why a large group of volunteers scores low on distress, but can also provide input for developing training courses and interventions aimed at increasing personal resources for volunteers with higher levels of distress.

## Data Availability Statement

The raw data supporting the conclusions of this article will be made available by the authors, without undue reservation.

## Ethics Statement

The studies involving human participants were reviewed and approved by ethical committee of the Faculty of Behavioral and Management studies (BMS) of University of Twente (approval number: 190943). The patients/participants provided their written informed consent to participate in this study.

## Author Contributions

RW and CD conceptualized the paper and drafted the paper. RW conducted all the statistical analyses. HM and EB revised the paper. All authors were involved in the design of the study and the construction of the questionnaire and final approval for the published article and agree to be accountable for all aspects of the work in ensuring that questions related to the accuracy or integrity of any part of the work are appropriately investigated and resolved.

## Conflict of Interest

The authors declare that the research was conducted in the absence of any commercial or financial relationships that could be construed as a potential conflict of interest.

## Footnotes

^1^For the readability, we only use the word “callers” here. Other users of the crisis line service, such as chatters and emailers, are also included.
